# Targeting noncanonical TGF-beta signaling: inhibition effects on the human keloid fibroblast transcriptome

**DOI:** 10.1097/itx.0000000000000084

**Published:** 2025-12-22

**Authors:** Jordan Morningstar, Athira Sivadas, Martin Romeo, Silvia Vaena, Manuel Valdebran, Shawn G. Kwatra, Russell A. Norris, Tyler C. Beck

**Affiliations:** a Department of Regenerative Medicine and Cell Biology, Medical University of South Carolina, Charleston, SC,; b Medical Scientist Training Program, Medical University of South Carolina, Charleston, SC,; c Vanderbilt University School of Medicine, Nashville, TN,; d Department of Biochemistry and Molecular Biology, Hollings Cancer Center, Medical University of South Carolina, Charleston, SC,; e Department of Dermatology and Dermatologic Surgery, Medical University of South Carolina, Charleston, SC,; f Department of Dermatology, University of Maryland, Baltimore, MD; g Department of Dermatology, Vanderbilt University Medical Center, Nashville, TN

**Keywords:** Pruritus, Keloids, Fibroblasts, MEK1, Transcriptomics, TGF-beta

## Abstract

Keloids are benign fibrotic lesions that frequently cause persistent itch and pain, yet their underlying molecular mechanisms remain poorly defined. Aberrant transforming growth factor-beta (TGF-β) signaling, particularly through noncanonical pathways such as mitogen-activated protein kinase (MAPK), has been implicated in keloid pathogenesis. In this study, we investigated the effects of selective MEK1 inhibition in vitro using NL350–02 on human keloid fibroblasts via bulk RNA sequencing. Treatment with NL350–02 induced substantial transcriptomic reprogramming, including downregulation of TGF-β receptors, IL-6, IL-17 receptors, and proinflammatory chemokines, alongside the upregulation of proapoptotic genes and marked suppression of proliferation (eg, > 3000-fold reduction in Ki-67 expression). Pathway analyses revealed significant enrichment in processes related to apoptosis, cytokine signaling, and TGF-β regulation. Despite paradoxical increases in certain collagen transcripts, the concurrent induction of apoptosis suggests an indirect antifibrotic mechanism. Furthermore, transcriptomic overlap with pruritic conditions and the downregulation of itch-related cytokines highlight MEK1 as a potential target for modulating neuroinflammation in keloid-associated itch. These findings suggest that MEK1 inhibition may offer a dual therapeutic benefit, attenuating both fibrosis and pruritus, and warrant further validation in preclinical keloid models.

Keloids are benign fibroproliferative skin tumors characterized by fibroblast proliferation with abnormal and excessive deposition of collagen that arise following cutaneous injury^[1]^. Keloids pose significant challenges in clinical management due to their tendency to recur, causing aesthetic and functional impairments^[2]^. In addition, keloids are often symptomatic, accompanied by itch and pain. Among these symptoms, itch is often cited by patients as one of the most distressing and persistent features of keloids, significantly impacting quality of life. Despite its prevalence, the molecular mechanisms underlying keloid-associated itch remain poorly understood. Current treatment options include surgical excision, corticosteroid and/or 5-fluorouracial injections, cryotherapy, laser therapy, radiation therapy, and silicone gel sheeting^[3]^. These approaches have limitations, and there remains a lack of consistently effective treatments with high recurrence. As a result, there is a need for the development of novel therapeutics with improved efficacy that target the underlying molecular mechanisms driving keloid formation and progression.

The transforming growth factor-beta (TGF-β) signaling pathway plays a crucial role in the pathogenesis of keloids^[4]^. TGF-β is a multifunctional cytokine involved in various cellular processes, including cell proliferation, migration, and extracellular matrix production. Canonical TGF-β signaling occurs through the activation of Smad proteins, leading to the transcriptional regulation of target genes. However, accumulating evidence suggests that noncanonical TGF-β signaling pathways, such as the mitogen-activated protein kinase (MAPK) and phosphoinositide 3-kinase (PI3K) pathways, also contribute to keloid development^[5,6]^. In keloid tissue, aberrant TGF-β signaling is observed, with increased expression and activation of TGF-β ligands and receptors^[4]^. This dysregulated signaling leads to excessive fibroblast proliferation and collagen synthesis, contributing to the characteristic fibrotic phenotype of keloids. In addition to driving fibrosis, aberrant TGF-β signaling may influence sensory nerve activity within keloid tissue, potentially contributing to pruritus. Crosstalk between fibroblasts and sensory neurons, mediated through cytokines and neuroactive factors, represents an emerging area of investigation. Specifically, noncanonical TGF-β signaling pathways, particularly the MAPK and PI3K pathways, have been implicated in enhancing fibroblast activation, migration, and extracellular matrix deposition in keloids^[6]^. Inhibitors specific to these pathways may have the potential to attenuate the aberrant fibroblast response observed in keloids. In this study, we aim to selectively target the noncanonical TGF-β signaling components in vitro and assess the resultant modulation of pathologic processes underlying keloid formation and associated symptoms.

## Methods

To examine the transcriptomic changes proceeding noncanonical TGF-β inhibition, we treated human dermal keloid fibroblasts with the selective low nanomolar range mitogen-activated protein kinase kinase 1 (MEK1) inhibitor NL350–02 (1 μM) and performed bulk RNA-sequencing^[7]^.

### Cell culture

Human keloid fibroblasts (Kel Fib; Catalog number: CRL-1762) were cultured according to the manufacturer’s protocol. Cells were cultured in Gibco Dulbecco’s modified Eagle medium (DMEM) (ATCC; No. 30–2002) using 10% fetal bovine serum and 1% penicillin-streptomycin. Cells were plated at a confluency of 5 × 10^5^ per 35 mm well 24 hours before treatment. For drug treatment, cells were serum starved and treated with vehicle or NL350–02 at 1 μM for 24 hours. Compounds were dissolved in 1% DMSO, and vehicle-treated cells received 1% DMSO in media. Following 24-hour treatment, cells underwent RNA isolation and were immediately stored at −80 °C until further use.

### RNA sequencing

Total RNA was extracted using the Promega Maxwell RSC automated instrument (Promega Corporation; catalog number: AS4500). Poly(A) enrichment was performed using the NEBNext Poly(A) mRNA Magnetic Isolation Module (New England Biolabs; catalog number: NEB #E3370). The NEBNext Ultra II Directional RNA Library Prep Kits for Illumina [New England Biolabs; catalog number: (NEB #E7765)] were used for the generation of RNA libraries. Data were deposited to Partek for analysis using a standard workflow as previously described^[8]^. Differential analysis was performed using DESeq2. Statistically significantly differentially expressed genes (FDR-step up: < 0.1) (4490 genes) were submitted to Advaita Bioinformatics for iPathway Analysis. All differential analyses employed a Bonferroni correction for multiple comparisons when assessing for statistical significance.

## Results

**Figure 1.**
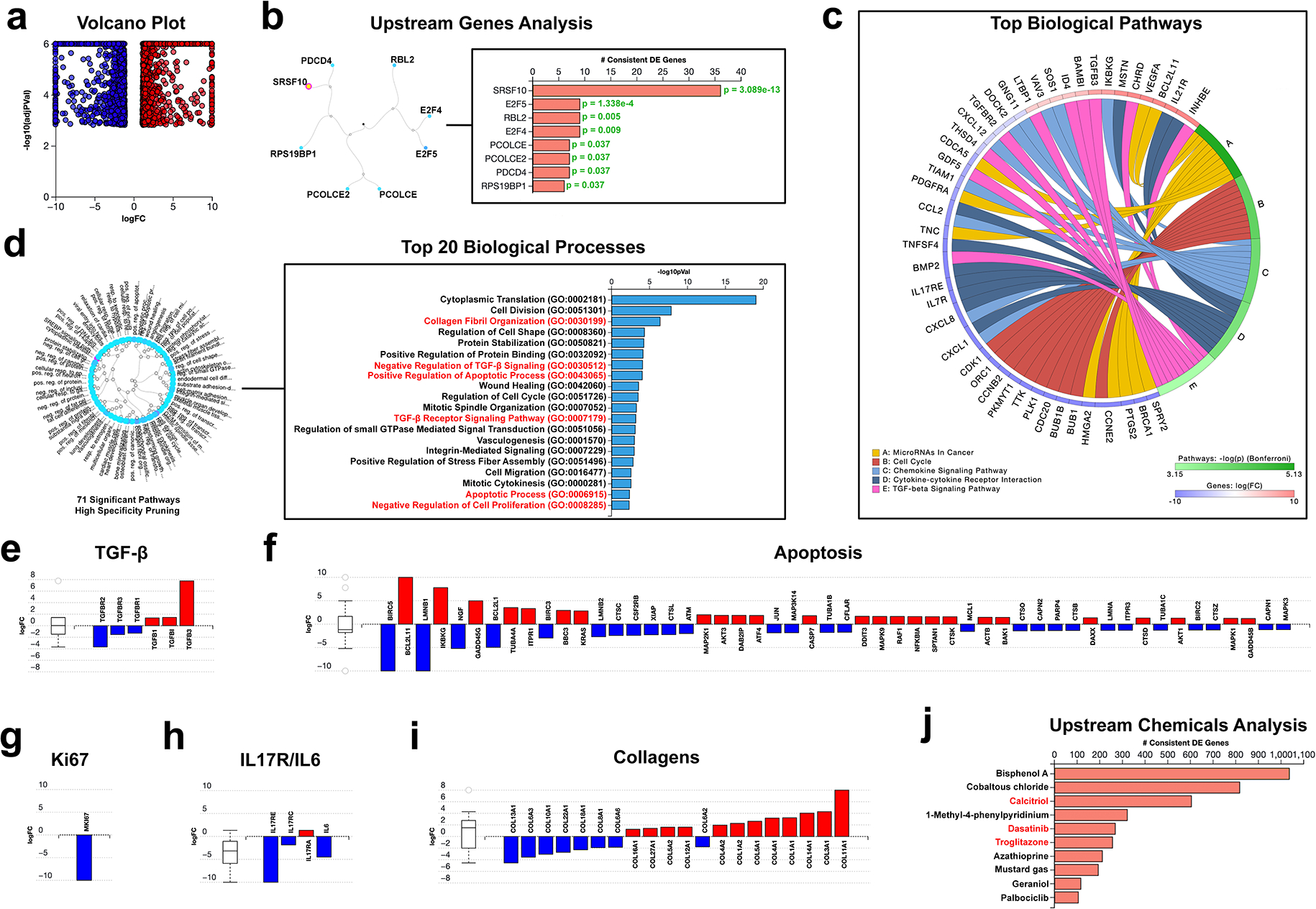
Analysis of the human keloid fibroblast transcriptome following MEK1 inhibition. A, Volcano plot: All 4490 significantly differentially expressed (DE) genes are represented in terms of their measured expression change (*x*-axis) and the significance of the change (*y*-axis). The significance is represented in terms of the negative log (base 10) of the *P* value, so that more significant genes are plotted higher on the *y*-axis. The upregulated genes (positive log fold-change) are shown in red, while the downregulated genes are shown in blue. B, Top upstream regulators predicted as activated. For each upstream regulator u, the table shows the number of DE targets supporting the hypothesis that the regulator is activated, DTA (u), the total number of DE genes downstream of u, DT(u), the combined raw *P* value, and the *P* value corrected for multiple comparisons. C, Top 5 biological pathways predicted to be activated in MEK1 inhibitor–treated keloid fibroblasts with the top 10 associated DEGs. D, Statistically significant divergent biological processes. Seventy-one biological processes were identified, with the top 20 displayed. Statistically significant DEGs relating to TGF-beta (E), apoptosis (F), Ki-67 (G), IL-17 and IL-6 signaling (H), and collagens (I). J, Upstream chemicals, drugs, toxicants (CDTs) predicted to be present based on the enrichment of DEGs and the network of interactions from the Advaita Knowledge Base (AKB v17.0). Statistical analyses were performed using a false discovery rate (FDR) *P* value correction (*P* ≥ 0.05).

In this experiment, 4490 differentially expressed genes (DEGs) were identified out of a total of 54,683 genes in Advaita Knowledge Base (AKB) (Fig. 1A). An upstream gene analysis was performed to identify and characterize upstream regulators promoting alterations in the keloid fibroblast transcriptome proceeding MEK1 inhibitor treatment. Of note, SRSF10 (serine/arginine-rich splicing factor 10) (*P* = 3.1e−13) and E2F5 (*P* = 1.338e−4) were top upstream regulators (Fig. 1B). SRSF10 participates in alternative splicing and can affect the expression of genes involved in apoptosis regulation^[9]^. E2F5 is a transcription factor involved in cell cycle regulation and cell proliferation, primarily functioning to repress the expression of genes required for cell cycle progression^[10]^. Top biological pathways affected by MEK1 inhibition included microRNAs in cancer (*P* = 7.382e−6), cell cycle (*P* = 6.286e−5), chemokine signaling (*P* = 9.999e−5), cytokine-cytokine receptor interaction (*P* = 1.518e−4), and TGF-β signaling (*P* = 7.123e−4) (Fig. 1C). High specificity pruning was employed to evaluate the top biological processes present within the data set. Seventy-one significant biological processes were identified: most notably, collagen fibril organization (*P* = 4.401e−7), negative regulation of TGF-β signaling (*P* = 8.935e−5), positive regulation of apoptotic process (*P* = 9.842e−5), TGF-β signaling (*P* = 7.041e−4), apoptotic process (*P* = 0.005), and negative regulation of cell proliferation (*P* = 0.005) (Fig. 1D). Of note, the expression of TGF-β receptors 1–3 was statistically reduced, whereas the expression of TGF-β ligands 1–3 was elevated (Fig. 1E and SDC, Figure 1, Supplemental Digital Content 1, http://links.lww.com/ITX/A23). Fifty-two DEGs consistent with a proapoptotic process were identified, with increased expression of Bak, Bim, PUMA, and ATF4, and decreased expression of Bcl-XL, IAP/XIAP, and FLIP (Fig. 1F and SDC, Figure 2, Supplemental Digital Content 1, http://links.lww.com/ITX/A23). Interestingly, expression of Ki-67, the gold-standard marker for cell proliferation, was reduced by 3000-fold, with a log fold-change of −10 (Fig. 1G). Both IL-17 and IL-6 contribute to the chronic inflammation observed in keloids and contribute to the fibrotic processes underlying their pathogenesis^[11]^. The expression of IL-17RE, IL-17RC, and IL-6 is reduced proceeding MEK1 inhibition, with downstream reduction in the expression of genes associated with an inflammatory response, such as AP-1, CXCL1, CXCL2, CXCL8, CCL2, and COX2 (Fig. 1H and SDC, Figure 3, Supplemental Digital Content 1, http://links.lww.com/ITX/A23). Paradoxically, expression of collagens globally increased, with increased expression of COL1A1, COL3A1, COL4A1, COL4A2, COL5A1, COL5A2, COL11A1, COL16A1, COL12A1, and COL27A1, and decreased transcription of COL6A2, COL6A3, COL6A6, COL8A1, COL10A1, COL13A1, COL18A1, and COL22A1 (Fig. 1I). These data challenge the notion that MEK1 inhibitors directly reduce the transcription of collagens but suggest that the antifibrotic effects of these drugs may be an indirect consequence of fibroblast cell death^[12]^. Lastly, an upstream chemicals analysis was performed to identify chemicals with related transcriptomic changes. Three out of the top 10 identified drugs have established preclinical efficacy in reducing keloid pathogenesis: calcitriol (*P* = 1.849e −20), dasatinib (*P* = 1.849e−20), and troglitozone (*P* = 1.849e −20) (Fig. 1J)^[13–15]^.

## Discussion

Our transcriptomic profiling of keloid fibroblasts treated with a selective MEK1 inhibitor reveals a multifaceted reprogramming of gene expression that supports the therapeutic potential of targeting noncanonical TGF-β signaling in keloid disease. MEK1 inhibition produced a robust proapoptotic and antiproliferative response, as evidenced by the upregulation of proapoptotic markers (Bak, Bim, PUMA, and ATF4) and the downregulation of proliferative markers, including a striking > 3000-fold decrease in Ki-67 expression. In addition, we observed the downregulation of TGF-β receptors 1–3 and a suppression of TGF-β downstream signaling, suggesting a feedback attenuation of fibrotic pathways.

Importantly, this perturbation also yielded anti-inflammatory transcriptional changes, with significant downregulation of IL-6, IL-17RC, and IL-17RE, and associated inflammatory mediators (CXCL1, CXCL2, CXCL8, CCL2, and COX2). These inflammatory signals are well-recognized contributors to pruritus, and their suppression provides a compelling link between MEK1 signaling and itch regulation in keloid pathology.

While MEK1 inhibition paradoxically increased the transcription of several collagen subtypes, including COL1A1, COL3A1, and COL5A2, this finding does not necessarily imply increased fibrotic deposition. In keloids, excess collagen accumulation and disorganized matrix are hallmarks of disease; however, the transcriptomic increases observed here are likely reflective of transient compensatory or stress-related transcription rather than sustained collagen synthesis. The concurrent induction of apoptosis and profound suppression of proliferative markers suggest that fibroblast viability and extracellular matrix production are globally reduced, consistent with an overall antifibrotic outcome. These data support the interpretation that the antifibrotic effects of MEK1 inhibition occur indirectly, through enhanced fibroblast apoptosis and reduced cellular proliferation, rather than through direct suppression of collagen gene transcription.

Moreover, the relationship between collagen expression and pruritus should be contextualized within disease-specific biology. In atopic dermatitis (AD), reduced expression of type I and III collagens contributes to impaired barrier integrity and enhanced neuronal sensitization, thereby promoting itch^[16]^. Conversely, in keloid disease, pruritus occurs in the setting of excessive collagen deposition and altered dermal architecture. Thus, the pathogenesis of itch in keloids is more plausibly driven by inflammatory and neuroimmune factors than by collagen abundance per se. The antipruritic potential of MEK1 inhibition, therefore, likely stems from the suppression of proinflammatory cytokines (IL-6, IL-17 family members, CXCL1, CXCL2, CXCL8, and CCL2) and downstream modulation of fibroblast-neuron crosstalk, rather than from changes in collagen subtype expression.

Collectively, these findings highlight a nuanced balance between proapoptotic signaling, altered collagen gene transcription, and attenuation of inflammatory cytokine output. MEK1 inhibition may simultaneously reduce fibroblast-driven fibrosis and dampen neuroinflammatory drivers of itch, offering a dual therapeutic benefit in keloid disease.

Clinically, MEK1 inhibitors (eg, trametinib, cobimetinib, binimetinib, and selumetinib) are already in use for aggressive malignancies, such as melanoma, and fibrotic tumors, such as plexiform neurofibromas. Our findings suggest a promising opportunity for repurposing MEK inhibitors as localized therapies (eg, topical or intralesional) for keloids. Such approaches may provide dual benefits by reducing both the physical lesion and the often-overlooked symptom of itch, which significantly affects patient quality of life. Current therapies largely fail to address pruritus, underscoring the need for mechanism-based interventions that target both fibrosis and neuroinflammation.

Finally, MEK1 inhibition modulated the expression of cytokines and chemokines (IL-6, IL-17, CXCL1, and CCL2) implicated in neuronal sensitization and itch transmission, suggesting that fibroblast-neuron crosstalk can be therapeutically modulated^[17–19]^. The observed transcriptomic overlap with pruritic disorders such as AD supports this hypothesis but also underscores the need for disease-specific interpretation of collagen changes. Future studies using spatial transcriptomics, fibroblast-sensory neuron cocultures, and in vivo xenograft or behavioral models should further clarify how MEK1 inhibition influences both fibrogenesis and pruritus in keloid disease. Functional validation of these pathways will be essential to confirm that MEK1 inhibition achieves a net antifibrotic and antipruritic effect through coordinated modulation of fibroblast survival, cytokine signaling, and extracellular matrix remodeling.

## Supplementary Material

Supplemental file

Supplemental Digital Content is available for this article. Direct URL citations are provided in the HTML and PDF versions of this article on the journal’s website, www.itch.com.

## Data Availability

The data used to generate the results in this study can be obtained from Vanderbilt University Medical Center and the Medical University of South Carolina (contact Tyler Beck: tyler.beck@vumc.org).
