# Supplementary material for: Targeting noncanonical TGF-beta signaling: inhibition effects on the human keloid fibroblast transcriptome
Source: Itch (Phila). Author manuscript; Available in PMC 2026 Apr 9. (PMC13061364; doi:10.1097/itx.0000000000000084)
Supplement: Supplemental file [file NIHMS2139051-supplement-Supplemental_file.docx]

**Targeting Non-Canonical TGF-Beta Signaling Pathway *In Vitro*: Inhibition Effects on the Human Keloid Fibroblast Transcriptome**

Tyler C. Beck, MD, PhD^1^

^1^ Department of Dermatology, Vanderbilt University Medical Center, Nashville, TN, USA

**SDC:**


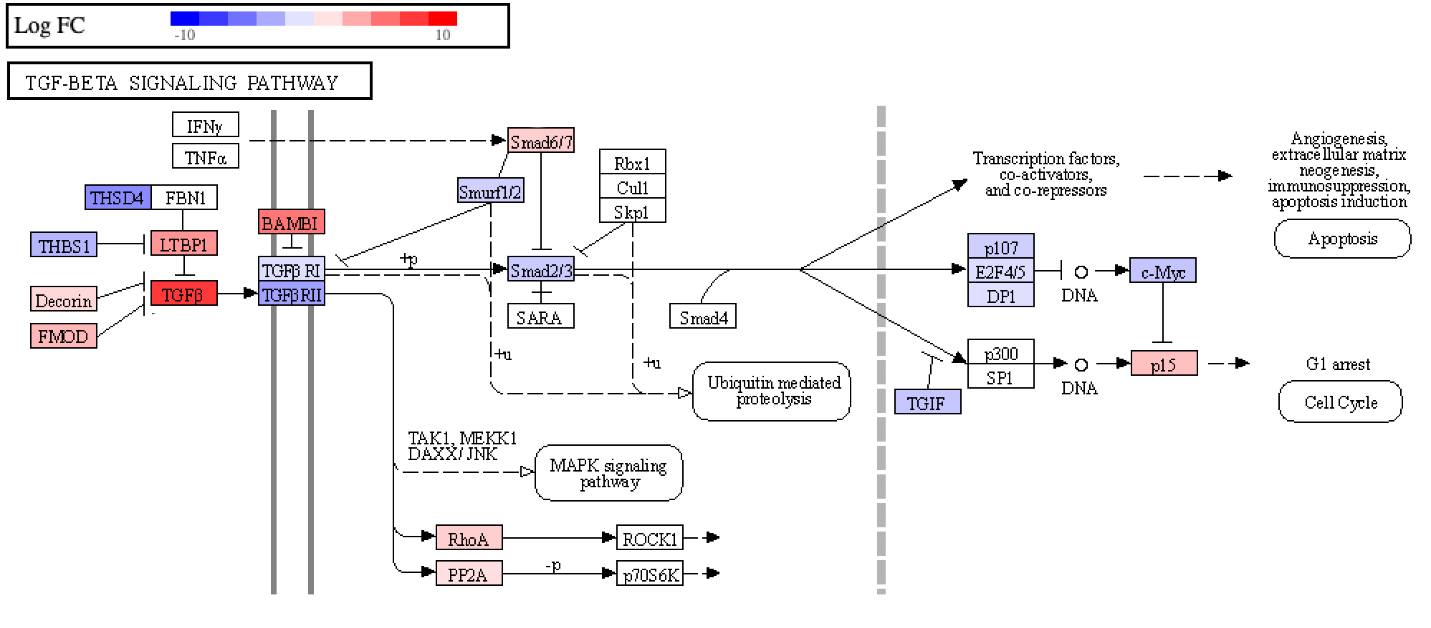
**SDC, Figure 1. TGF-beta signaling pathway.** The pathway diagram is overlayed with the computed perturbation of each gene. The perturbation accounts both for the gene's measured fold change and for the accumulated perturbation propagated from any upstream genes (accumulation). The highest negative perturbation is shown in dark blue, while the highest positive perturbation in dark red. The legend describes the values on the gradient. TGF-beta and BAMBI showed the most significant positive perturbation. Other downstream genes including Smad 6 and 7, RhoA, Decorin, FMDD, PP2A, and pI5 showed slight positive perturbation. Genes showing negative perturbation include THSD4, THBS1, TGF-beta RI, TGF-beta RII, Smad 1 and 2, Smurf 1 and 2, TGIF, DPI, E2F4, E2F5, pI07, and c-Myc. Overall, signaling had a net pro-apoptotic affect and promoted cell cycle arrest.


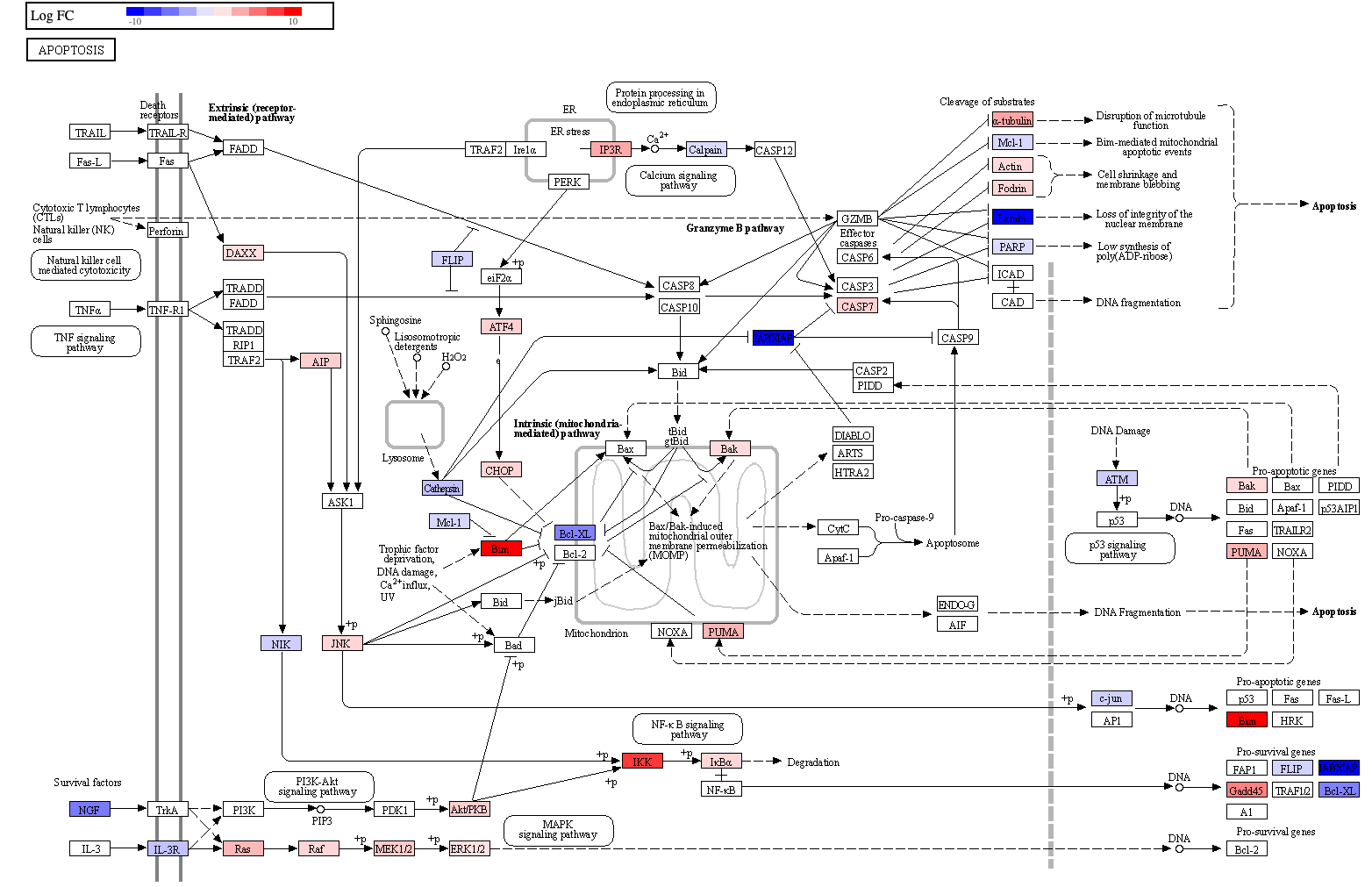


**SDC, Figure 2. Apoptosis.** The pathway diagram is overlayed with the computed perturbation of each gene. The perturbation accounts both for the gene's measured fold change and for the accumulated perturbation propagated from any upstream genes (accumulation). The highest negative perturbation is shown in dark blue, while the highest positive perturbation in dark red. The legend describes the values on the gradient. Pro-apoptotic genes: IKK and Bim showed the most significant positive perturbation. Other pro-apoptotic genes such as: Akt/PKB, I-kappa-B-alpha, PUMA, Bak, CASP9, Fordrin, actin, alpha-tubulin, IP3R, DAXX, AIP, CHOP, ATF4, and JNK, showed slight positive perturbation. Some pro-apoptotic genes had negative perturbation including Bcl-XL, c-jun, NIK, FLIP, cathepsin, Mcl-l, calpain, IAP/XIAP, Lamin, and PARP. A number of Pro-survival genes also showed positive perturbation including: Gadd45, Ras, Raf, Mek1/2, ERK1/2; while other pro-survival genes showed negative perturbation: IAP/XIAP, FLIP, Bcl-XL, NGF, and IL-3R.


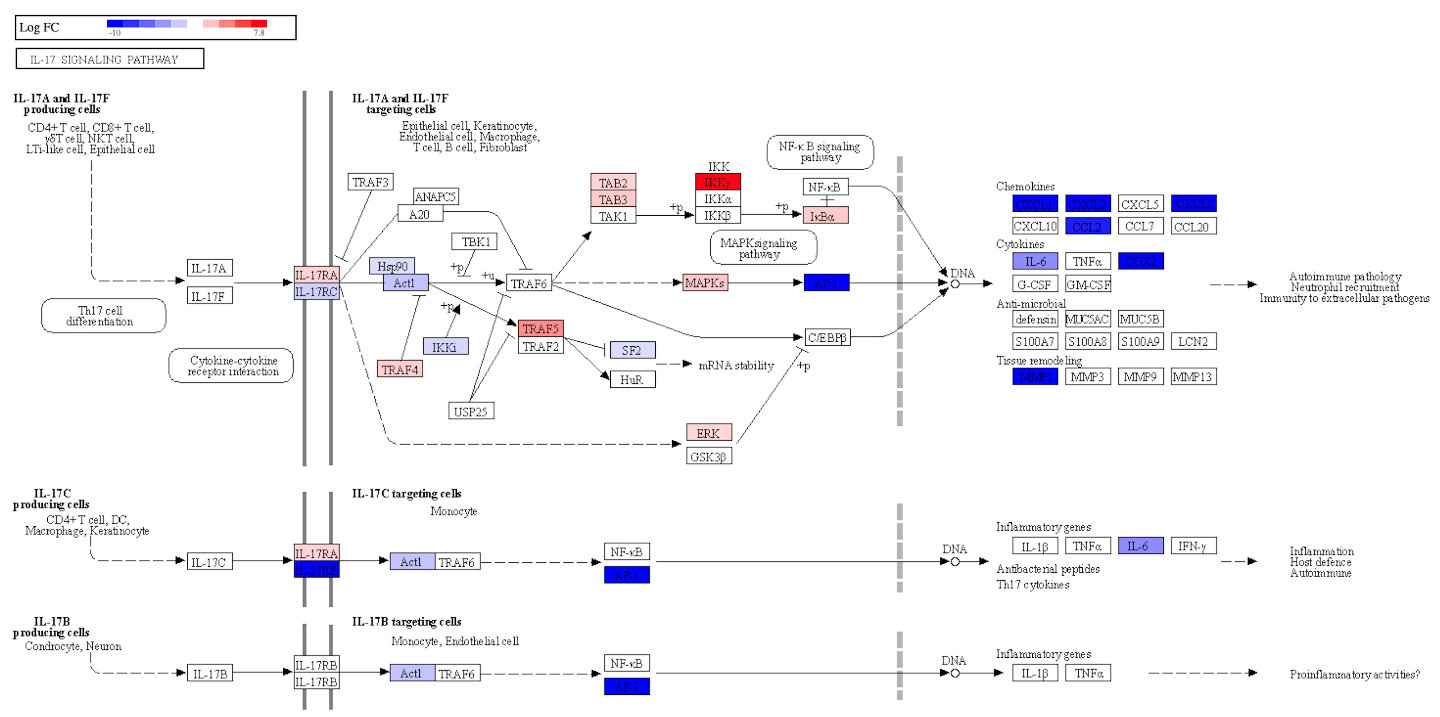
**SDC, Figure 3. IL-17 Signaling Pathway.** The pathway diagram is overlayed with the computed perturbation of each gene. The perturbation accounts both for the gene's measured fold change and for the accumulated perturbation propagated from any upstream genes (accumulation). The highest negative perturbation is shown in dark blue, while the highest positive perturbation in dark red. The legend describes the values on the gradient. Several genes in the IL-17 signaling pathway showed positive perturbation including: IL-17RA, TRAF4, TRAF5, TAB2, TAB3, IKK-gamma, MAPKs, ERK, and I-kappa-B- alpha, with IKK-gamma being the most significant. However, multiple genes showed significant negative perturbation including IL-17RE, AP-1, Act1, IL-6, MMP1, COX2, CXCL1, CXCL2, CXCL3, CCL2, HSP90, IL- 17RC, SF2, and IKKi.
